# Medium-term health and social outcomes in adolescents following sexual assault: a prospective mixed-methods cohort study

**DOI:** 10.1007/s00127-021-02127-4

**Published:** 2021-08-09

**Authors:** Venetia Clarke, Andrea Goddard, Kaye Wellings, Raeena Hirve, Marta Casanovas, Susan Bewley, Russell Viner, Tami Kramer, Sophie Khadr

**Affiliations:** 1 The Havens Sexual Assault Referral Centres, King’s College Hospital, Denmark Hill, London, SE5 9RS UK; 2 Department of Paediatrics, Imperial College Healthcare NHS Trust, London, W2 1NY UK; 3 Faculty of Public Health and Policy, London School of Hygiene and Tropical Medicine, Keppel Street, London, WC1E 7HT UK; 4 Department of Women and Children’s Health, King’s College London, c/o 10th Floor North Wing, St Thomas’ Hospital, Westminster Bridge Road, London, SE1 7NH UK; 5 Division of Psychiatry, Imperial College London, 7th Floor Commonwealth Building, Du Cane Road, London, W12 0NN UK; 6 Great Ormond Street Institute of Child Health, University College London, 30 Guilford Street, London, WC1N 3EH UK

**Keywords:** Adolescent, Rape, Sexual assault, Mental health, Education, Longitudinal cohort study

## Abstract

**Purpose:**

To describe medium-term physical and mental health and social outcomes following adolescent sexual assault, and examine users’ perceived needs and experiences.

**Method:**

Longitudinal, mixed methods cohort study of adolescents aged 13–17 years recruited within 6 weeks of sexual assault (study entry) and followed to study end, 13–15 months post-assault.

**Results:**

75/141 participants were followed to study end (53% retention; 71 females) and 19 completed an in-depth qualitative interview. Despite many participants accessing support services, 54%, 59% and 72% remained at risk for depressive, anxiety and post-traumatic stress disorders 13–15 months post-assault. Physical symptoms were reported more frequently. Persistent (> 30 days) absence from school doubled between study entry and end, from 22 to 47%. Enduring mental ill-health and disengagement from education/employment were associated with psychosocial risk factors rather than assault characteristics. Qualitative data suggested inter-relationships between mental ill-health, physical health problems and disengagement from school, and poor understanding from schools regarding how to support young people post-assault. Baseline levels of smoking, alcohol and ever drug use were high and increased during the study period (only significantly for alcohol use).

**Conclusion:**

Adolescents presenting after sexual assault have high levels of vulnerability over a year post-assault. Many remain at risk for mental health disorders, highlighting the need for specialist intervention and ongoing support. A key concern for young people is disruption to their education. Multi-faceted support is needed to prevent social exclusion and further widening of health inequalities in this population, and to support young people in their immediate and long-term recovery.

**Supplementary Information:**

The online version contains supplementary material available at 10.1007/s00127-021-02127-4.

## Introduction

Sexual violence represents a major global public health problem [1, 2], with young people significantly at risk [2, 3], particularly if socially disadvantaged or with pre-existing vulnerabilities [2, 4–7]. Interpersonal violence and child sexual abuse are leading risk factors for disability-adjusted life-years and mortality among adolescents [8], yet there remain many gaps in understanding about the longer-term impacts of sexual violence in childhood and adolescence and how services should respond to these needs [9].

Sexual violence during adolescence is associated with negative functional outcomes including poor academic performance, sexual risk-taking behaviour, pregnancy, substance abuse, and interpersonal problems [3, 10–14]. Little is known about the causal direction of these associations or the level and trajectory of mental health and social outcomes. The few existing prospective studies show a high proportion of acute sexual assault victims have short-term adverse mental health outcomes [15, 16]. Non-prospective studies suggest that adolescent victims of sexual violence are at increased risk of depression, post-traumatic stress disorder (PTSD), anger control problems, low self-esteem, eating-disordered behaviour, and suicidality, compared to non-affected peers [3, 11, 12, 17–19].

Adolescence is a time of rapid brain development and biopsychosocial change [20, 21] with the potential for adverse experiences to have a profound impact on social and emotional functioning. Adverse experiences during adolescence have been associated with increased psychological distress at age 18 [22]. The World Health Organisation has highlighted the need for more longitudinal research to inform the long-term needs of and support provided to child and adolescent survivors of sexual violence [9]. Many mental health sequalae described in the adult literature present early and respond well to treatment if addressed, but if untreated may persist and recur with implications for long-term functioning [23, 24].

The purpose of this mixed-methods study is to illustrate the nature and trajectory of medium-term health and social outcomes following adolescent sexual assault to inform interventions to minimise harm and promote recovery, healthy functioning and development.

## Methods

This prospective mixed-methods cohort study of adolescents experiencing sexual assault in a large urban area (London, UK) was conducted according to a pre-specified protocol (Haven Study Protocol v1.5 2nd September 2013, Institute of Child Health, 4^th^ February 2017 https://www.ucl.ac.uk/ich/research/population-policy-practice/research/studies/adolescent-sexual-assault) aligned to STROBE reporting guidelines. The primary and early secondary outcomes have been fully reported previously [15]. This paper reports on medium-term secondary outcomes including: education and employment status; physical and mental health (including substance use); uptake of mental health services and other health service use since assault; and participants’ evaluation of needs post-assault. Judicial outcomes and user experience of services are the focus of another paper.

### Setting

The Havens are specialist sexual assault referral centres providing forensic medical examinations and follow-on medical and psychological support in Greater London, UK. They are jointly funded by the National Health Service and the Mayor’s Office for Policing and Crime. Referrals are from the police (85–90% of cases), other statutory and voluntary sector (non-governmental, non profit) services, or individuals may self-refer.

### Population and recruitment

The study recruited 141 adolescents (134 female, 6 male, and 1 transgender young woman) aged 13–17 years inclusive within 6 weeks of a sexual assault [15]. The initial baseline sample (29% participation) was broadly representative of all those eligible to participate who presented to the Havens [15]. Participants had a baseline assessment at study entry. Follow-up assessments were conducted 4–5 months post-assault (short-term and primary outcome) and at study end, 13–15 months post-assault (medium-term and secondary outcomes, 12–14 months post recruitment).

Information collected at study entry and end included: physical and mental health symptoms; health service access; substance use; and social outcomes (education and employment status). Participants were then invited to take part in a qualitative interview as outlined below.

### Quantitative data collection

#### Socio-demographic and assault characteristics

Data collection has been described previously [15], including use of the Index of Multiple Deprivation (IMD) as a measure of area deprivation [25]. Socio-demographic and assault characteristics of study completers were compared with entrants to assess associations with retention (see Table 1).

**Table 1:** Baseline demographic, psychosocial and assault-related characteristics of the cohort at study start and for those retained at study end

	All adolescents recruited						
	Study start (*n* = 141)		Study end (*n* = 75)		*P* value	Difference	95% CI (%)
	*N*^a^	*n* (%)	*N*^a^	*n* (%)			
Gender					1.000		
Female	141	134 (95.0%)	75	71 (94.7%)		− 0.3%	− 6.1, 9.3
Male	141	6 (4.3%)	75	3 (4.0%)		− 0.3%	− 8.2, 6.2
Transgender female	141	1 (0.7%)	75	1 (1.3%)		0.6%	
Age at assault							
Mean (SD) age	141	15.59 (1.27)	75	15.6 (1.35)	0.331		− 1.2, 0.6
13–15 years	141	87 (61.7%)	75	43 (57.3%)	0.299	− 4.4%	− 9.7, 18.7
16–17 years	141	54 (38.3%)	75	32 (42.7%)			
Ethnicity					0.124		
White	141	72 (51.1%)	75	33 (44.0%)		− 7.1%	− 7.6, 21.2
Black	141	40 (28.4%)	75	27 (36.0%)		7.6%	− 5.8, 21.6
South Asian	141	4 (2.8%)	75	2 (2.7%)		− 0.1%	− 7.6, 5.4
Mixed	141	23 (16.3%)	75	11 (14.7%)		− 1.6%	− 10.2, 11.7
Other	141	2 (1.4%)	75	2 (2.7%)		1.3%	− 3.4, 8.9
Index of multiple deprivation (IMD)^b^ at baseline					**0.034**		
IMD Deciles 1–2 (most deprived)	141	49 (34.8%)	75	21 (28.0%)		− 6.8%	− 7.3, 19.5
IMD Deciles 3–4	141	53 (37.6%)	75	30 (40.0%)		2.4%	− 11.5, 16.8
IMD Deciles 5–6	141	23 (16.3%)	75	18 (24.0%)		7.7%	− 3.8, 20.4
IMD Deciles 7–8	141	9 (6.4%)	75	4 (5.3%)		− 1.1%	− 8.0, 7.8
IMD Deciles 9–10 (least deprived)	141	7 (5.0%)	75	2 (2.7%)		− 2.3%	− 5.7, 8.1
Living with at baseline					0.172		
Both parents	141	33 (23.4%)	75	18 (24.0%)		0.6%	− 11.3, 13.8
1 parent	141	71 (50.4%)	75	42 (56.0%)		5.6%	− 9.0, 19.8
Other living arrangement^c^	141	37 (26.2%)	75	15 (20.0%)		− 6.2%	− 6.9, 17.7
Education or employment status pre-assault							
Attending school	138	119 (86.2%)	75	63 (84.0%)	0.464	− 2.2%	− 7.9, 14.1
Non-mainstream (special) school or unit^d^	119	20 (16.8%)	63	9 (14.3%)	0.470	− 2.5%	− 10.6, 13.4
Employed	140	15 (10.7%)	75	9 (12.0%)	0.785	1.3%	− 7.7, 12.3
Not in education or employment	138	16 (11.6%)	75	9 (12.0%)	1.000	0.4%	− 8.7, 11.5
Psychosocial Characteristics at baseline							
Current/previous extra help with learning at school (1:1 or small group)	138	52 (37.7%)	73	28 (38.4%)	1.000	0.7%	− 13.3, 15.2
Current/previous statement of Special Education Needs (SEN)	132	25 (18.9%)	72	12 (16.7%)	0.509	− 2.2%	− 10.3, 13.1
Mental health help in the 12 months prior to the assault^e^	140	71 (50.7%)	75	34 (45.3%)	0.180	− 5.4%	− 9.2, 19.6
Self-harm in the 12 months prior to the assault	140	57 (40.7%)	74	27 (36.5%)	0.305	− 4.2%	− 10.3, 17.9
Social Services involvement prior to or at the time of the assault	132	68 (51.5%)	72	28 (38.9%)	**0.002**	− 12.6%	− 2.5, 26.7
Ever in foster care prior to or at the time of the assault	137	27 (19.7%)	74	13 (17.6%)	0.525	− 2.1%	− 10.4, 13.0
Previous sexual abuse or assault	136	43 (31.6%)	74	25 (33.8%)	0.583	2.2%	− 11.3, 16.4
History of running away	138	55 (39.9%)	75	25 (33.3%)	0.116	− 6.6%	− 7.9, 19.9
Ever used alcohol	124	104 (83.9%)	70	56 (80.0%)	0.223	− 3.9%	− 7.6, 16.8
Binge drinking on at least one occasion in the last month^f^	116	34 (29.3%)	66	16 (24.2%)	0.217	− 5.1%	− 9.6, 18.3
Ever use of recreational drugs	140	63 (45.0%)	74	31 (41.9%)	0.497	− 3.1%	− 11.5, 17.2
Referral Pathway							
Police referral	141	128 (90.8%)	75	69 (92.0%)	0.772	1.2%	− 8.8, 9.1
Assailant Characteristics							
Stranger assault	133	51 (38.3%)	72	29 (40.3%)	0.721	2.0%	− 12.3, 16.6
Multiple assailant assault	137	29 (21.2%)	74	17 (23.0%)	0.676	1.8%	− 10.0, 14.9
Assailant(s) aged ≤ 20 years^g^	121	76 (62.8%)	61	41 (67.2%)	0.350	4.4%	− 11.4, 18.9
Assailant(s) aged > 20 years^g^	121	48 (39.7%)	60	21 (35.0%)	0.354	− 4.7%	− 11.4, 19.5
Assault characteristics							
Substance use around the time of the assault	125	47 (37.6%)	66	20 (30.3%)	0.096	− 7.3%	− 7.9, 21.1
Violent assault (weapons or physical violence)	114	63 (55.3%)	65	39 (60.0%)	0.260	4.7%	− 11.1, 19.9
Rape	126	116 (92.1%)	72	67 (93.1%)	0.744	1.0%	− 9.0, 8.9
Vaginal rape	119	97 (81.5%)	67	52 (77.6%)	0.242	− 3.9%	− 8.3, 17.6
Anal rape	117	19 (16.2%)	69	14 (20.3%)	0.205	4.1%	− 7.7, 17.2
Oral rape	122	52 (42.6%)	71	30 (42.3%)	1.000	− 0.4%	− 14.8, 15.1
> 1 type of rape	121	46 (38.0%)	70	25 (35.7%)	0.573	− 2.3%	− 12.8, 16.6
Penetration, digital or by an object	110	38 (34.5%)	63	21 (33.3%)	0.840	− 1.2%	− 14.5, 16.0
Other sexual assault^h^	112	73 (65.2%)	60	34 (56.7%)	**0.049**	− 8.5%	− 7.3, 24.4
Type of assault not known or no recollection of events	141	13 (9.2%)	75	3 (4.0%)	**0.038**	− 5.2%	− 3.8, 12.2

*P* values < 0.05 were considered significant and are in bold

Significance tests: Independent samples *T* Test for continuous data. Chi Square or Fisher's Exact Test (if any expected cell counts were under 5) for categorical data

Confidence intervals: http://www.vassarstats.net/prop2_ind.html

^a^Denominator is all those from the sample with available data unless otherwise specified

^b^[25]

^c^Other living arrangements include in foster care, living independently, with friends or other family

^d^Denominator is those attending school

^e^Options included General Practitioner (GP), mental health professional, counsellor, other health professional (e.g., Emergency Department), Social Services or voluntary agency

^f^5 or more drinks on one occasion[32]

^g^Takes into account single and multiple assailant assaults

^h^Other sexual assault included: touching, kissing, biting, cunnilingus, attempted rape/penetration, fellatio or vaginal sex forced on male victim

#### Mental health symptoms, self-harm, substance use, and related service access

Mental health symptoms were assessed at study entry [15] and end using: the Strengths and Difficulties Questionnaire (SDQ), a measure of psychosocial problems and strengths [26]; the Child Revised Impact of Events Scale (CRIES) [27] for post-traumatic stress (PTS) symptoms; the Short Mood and Feelings Questionnaire (SMFQ) [28] for depressive symptoms; and the Screen for Child Anxiety Related Disorder (SCARED) [29] for anxiety. Smoking, alcohol and illicit drug use were measured using age-appropriate questions derived from national surveys [30–32]. At entry, participants were asked about self-harm and about services accessed for mental health help in the 12 months prior to assault. At follow-up assessments, they were asked about self-harm and about any services accessed during the intervening period.

#### Physical symptoms and related service access

At study entry, participants were asked about physical symptoms and health services accessed in the 12 months prior to assault. At follow-up assessments, they were asked about physical symptoms and about health services accessed during the intervening period.

#### Social outcomes

Education or employment status was assessed at study entry and end. Participants in school were also asked about prolonged absence (> 30 days in the last 12 months) at both time points. Those with prolonged absence from school, and those not in education, employment or training (NEET) were defined as disengaged from education and employment. Other social outcomes examined included revictimization (since the assault) and (cumulative) prevalence of ever being in foster care by study end.

### Qualitative data collection

All participants attending the study end follow-up assessment were invited to express their interest in taking part in a further in-depth, semi-structured face-to-face interview. A purposive sample was recruited reflecting the age range, ethnic diversity, gender mix, pre-existing vulnerability and assault characteristics of the study cohort, with additional written consent sought. Interviews were conducted by two members of the study team, guided by a topic guide at a venue offering confidentiality, of the participant’s choice. With participants’ permission, interviews were audio-recorded using an encrypted digital recorder and the recordings were transcribed verbatim. Areas covered in the interviews included help-seeking following the assault; perceived impact of the assault on participation in work and education, mental and physical health, and relationships with family members; and the source, extent and quality of support subsequently received.

### Analyses

Analyses were undertaken using SPSS 25 (IBM Analytics) or STATA 16 (StataCorp, College Station TX). T tests were used to compare continuous variables and Chi squared, McNemar or Fisher’s exact tests were used for categorical variables.

Characteristics of the study cohort were described using simple statistics, comparing the profiles of those recruited with those retained at study end. Evaluation of mental health symptom questionnaire data was limited to full scales rather than subscales to reduce statistical comparisons. Longitudinal changes in mental health symptoms, substance use and education or employment status and changes in self-harm, physical symptoms and health service use since the assault were assessed.

#### Missing data 

For individual variables, missing data are shown via a change in denominator. Total scores were calculated and reported even for those with missing data; the exception to this was exclusion of SCARED questionnaire data for two participants with > 30% missing data at study entry and/or end. Following sensitivity analyses, all other missing item responses in the S-MFQ, SCARED and CRIES were treated as negative, thereby producing conservative estimates of symptoms. SDQ scores were generated in SPSS or Stata using standardised scoring syntax [33, 34].

#### Logistic regression

Further statistical analysis was undertaken for females only due to 95% preponderance. Logistic regression was used to examine associations between key outcomes at study end [(i) mental health symptoms above threshold (suggesting increased likelihood of disorder) and (ii) disengagement with education or employment], and a number of potential predictors: baseline demographic factors (age, ethnicity and deprivation); assault characteristics; baseline and follow-up vulnerability indicators; mental health disorder at first follow-up (primary outcome) [15] and mental health help accessed prior to or since the assault. Odds ratios are shown unadjusted and adjusted for age, ethnicity and deprivation.

#### Qualitative analysis

Data from the qualitative interviews were analysed using ‘Framework’, a content analysis method of proven validity and reliability and which allows systematic within-and cross-case analysis. [35]. This method was chosen for its suitability for applied policy research in which specific questions to be answered have already been identified. Interview transcripts were reviewed for initial and emergent themes by two team members, coded and any disagreements discussed to improve the reliability of coding. Coding was largely deductive, that is, codes were selected for their specific relevance to the study aims, but open coding was additionally used to ensure that significant areas of experience were not missed. Interview data were summarized in a matrix, and interpretative analyses of the charted data and themes were carried out using the constant comparative method [36]. Findings from the qualitative work were used to illuminate, contextualize and deepen understanding of the quantitative findings, where possible [37].

#### Ethical considerations

The study was approved by the National Research Ethics Service Oxford A Committee on 14th March 2013 (ref no. 12/SC/0339). All participants gave informed consent prior to inclusion. Participants in the qualitative interviews gave informed consent to use of anonymised quotations in publications. All participants were invited to a private dissemination event and several subsequently participated in two public events.

## Results

### Sample characteristics and congruence

Seventy-five adolescents were followed up (53% retention). Study end assessments took place at a median (IQR) of 459 (430–488) days, or 15.3 (14.3–16.3) months post assault.

The cohort was 95% female (71 female, 3 male and 1 transgender young woman), 44% white, and had a mean age of 15.6 years at assault. The majority (92%) had attended the Havens as police referrals. Two thirds (68%) lived in the two most deprived IMD area quintiles [25]. One in six (18%) and two in five (39%) had a history of being in foster care or of previous social services involvement, respectively, prior to or at the time of the assault. One in three (33%) had a history of running away. One in six had a statement of special educational needs (17%) and 38% had extra help at school, with 14% of those in school attending a special school or unit. Twelve percent (12%) were neither in education, or employed at study entry. A third (34%) disclosed previous sexual abuse or assault. In a sizeable proportion, the index assault involved strangers (40%), multiple assailants (23%), violence (weapons or physical violence in 60%), or substance use around the time (30%). Ninety-three percent (93%) of assaults were reported rape.

Nineteen qualitative interviews (17 female, 1 male and 1 transgender young woman) were conducted at a median (range) of 43 (6–70) days following the study end assessment.

Table 1 shows the largely congruent characteristics of the cohort at recruitment and study end, for all participants. A smaller proportion of adolescents completing the study had social services involvement prior to the assault (39% vs. 52%, *p* = 0.002), and there were differences in IMD area distribution (*p* = 0.034).

### Medium-term health outcomes and service access

Tables 2 and 3 show medium-term health outcomes for all participants following sexual assault, including longitudinal change in mental health symptom levels and substance use (Table 2), and changes in self-harm, physical symptoms and health service use (Table 3). Female-only data can be found in Supplementary Material. The proportion of young people reporting anxiety, PTS or depressive symptom levels above threshold (suggesting disorder) decreased significantly over time. However, 13–15 months post assault, 60%, 72% and 54%, respectively, remained at risk for an anxiety disorder, PTSD or depression. In-depth interview participants gave vivid descriptions of their states of mind, describing feelings of worthlessness, withdrawal, stress, anger and anhedonia. Some were unable to leave their homes. Normal functioning was impaired, sleep was disturbed, and panic attacks were common:

“[…] I’d literally become violent with rage and anger, […] and I remember kicking someone in their head, […] and I wasn’t a violent person, it’s just the minute someone said the word rape […]”

“I was just really down, really, really down, […] at the time I didn’t have nothing that I enjoyed, there was nothing.”

“… my anxiety was really bad […] Your heart beats fast […], and you get really sweaty and stuff, you know. It’s like you just can’t talk, you know, you can’t physically move, it’s like you’re frozen.”

**Table 2:** Longitudinal changes in mental health symptoms and substance use

	All participants followed up to study end (n = 75)				
	*N*^a^	Study entry	Study end	*P* value*	% diff.
		*n* (%)	*n* (%)		
Mental Health Symptom score ≥ threshold					
CRIES-13^b^	71	64 (90.1%)	51 (71.8%)	**0.004**	18.3%
S-MFQ^c^	72	64 (88.9%)	39 (54.2%)	** < 0.000**	34.7%
SCARED^d^	67	51 (76.1%)	40 (59.7%)	**0.019**	16.4%
SDQ: total score^e^	70	24 (34.3%)	16 (22.9%)	0.057	11.4%
Substance use					
Current smoker	72	26 (36.1%)	33 (45.8%)	0.143	− 9.7%
Current alcohol use	73	45 (61.6%)	58 (79.5%)	**0.002**	− 17.8%
Binge drinking in last month	65	16 (24.6%)	17 (26.2%)	1.000	− 1.5%
Drunk in the last month	68	16 (23.5%)	19 (27.9%)	0.648	− 4.4%
Ever used drugs (at study entry)	74	31 (41.9%)	–	–	–
Ever used drugs by study end (cumulative measure)	69	–	42 (60.9%)	–	–

*McNemar Test. *P* values < 0.05 were considered significant and are in bold

^a^N = number with valid data

^b^Child Revised Impact of Events Scale; cut-off score of ≥ 30;

^c^Short Moods and Feelings Questionnaire; cut-off score of ≥ 8;

^d^Screen for Child Anxiety Related Disorder; cut-off score of ≥ 30, cases with ≥ 30% missing data excluded;

^e^Strengths and Difficulties Questionnaire; cut-off score of ≥ 20

**Table 3:** Changes in self-harm, physical symptoms and health service use following sexual assault

	All participants followed up to study end (n = 75)				
	*N*^a^	In 12 months prior to assault	Post assault^b^	*P* value*	% diff.
		*n* (%)	*n* (%)		
Self-harm	69	26 (37.7%)	35 (50.7%)	0.108	− 13.0%
Physical symptoms					
Headaches	70	40 (57.1%)	51 (72.9%)	0.520	− 15.7%
Abdominal pain	65	29 (44.6%)	40 (61.5%)	**0.043**	− 16.9%
Poor sleep	71	33 (46.5%)	62 (87.3%)	** < 0.000**	− 40.8%
Changes in appetite	68	18 (26.5%)	51 (75.0%)	** < 0.000**	− 48.5%
Other physical symptoms	64	1 (1.6%)	4 (6.3%)	0.375	− 4.7%
Any physical symptoms	74	54 (73.0%)	72 (97.3%)	** < 0.000**	− 24.3%
Health service visit for physical symptoms					
Visited a general practitioner (GP)	68	37 (54.4%)	48 (70.6%)	0.052	− 16.2%
Visited a hospital	62	27 (43.5%)	31 (50.0%)	0.523	− 6.5%
Visited other service	64	8 (12.5%)	13 (20.3%)	0.302	− 7.8%
Visited any service	71	46 (64.8%)	58 (81.7%)	**0.012**	− 16.9%
Health service visit for mental health symptoms					
GP	65	13 (20.0%)	12 (18.5%)	1.000	1.5%
Mental health professional^c^	68	20 (29.4%)	41 (60.3%)	** < 0.000**	− 30.9%
Counsellor^d^	71	19 (26.8%)	30 (42.3%)	0.052	− 15.5%
Other medical professional^e^	65	1 (1.5%)	5 (7.7%)	0.219	− 6.2%
Other service^f^	68	2 (2.9%)	12 (17.6%)	**0.006**	− 14.7%
Any service accessed for mental health help	73	34 (46.6%)	58 (79.5%)	** < 0.000**	− 32.9%
More than 1 service accessed	73	19 (26.0%)	29 (39.7%)	0.087	− 13.7%

*McNemar Test. *P* values < 0.05 were considered significant and are in bold

^a^*N* = number with valid data

^b^Post assault: symptoms reported at first follow-up (4–5 months post sexual assault) and/or second follow-up (study end,13–15 months post sexual assault)

^c^Mental health professional: Child and Adolescent Mental Health Services (CAMHS), family therapy, adult mental health services, psychologist, psychiatrist, psychotherapy. Includes in-and out-patient care

^d^Counsellor: unspecified counselling, school counsellor, grief/bereavement counselling, group counselling, behavioural counselling, counselling for individuals who have suffered abuse

^e^Other medical professional: Accident and Emergency (A&E), school nurse, gynaecology, sexual health clinic

^f^Other service: social services, school, voluntary sector support, advocacy, alternative therapies and unspecified support

Baseline levels of smoking, alcohol use and ever drug use (the latter measured cumulatively) were high at recruitment and higher at study end (36% vs 46%, 62% vs 80%, and 42% vs 61%, respectively), although only the alcohol use increase was significant. Levels of binge-drinking did not change overall (25% vs. 26%), but there were changes in individuals’ behaviour, with 12% stopping and 14% starting anew. The qualitative research confirmed the use of recreational drugs and alcohol to distract from unwanted feeling or thoughts or to help with sleep problems.

“I was smoking like two [£]20 bags worth of weed to get so high […] I didn’t know any other way to filter out the feelings, so I was just smoking, smoking, smoking […]”

“Drink, that’s another thing that helps me sleep, that’s the main reason that I do it half the time, just to sleep, […]”

Self-harm was common in the 12 months preceding sexual assault and reported more frequently afterwards (38% vs. 51%). A quarter of participants (25%) started self-harming after the assault. Twenty-six percent (26%) had also self-harmed previously, with evidence from the qualitative data suggesting that its practice was resumed as a means of coping.

“I used to cut. […] I don’t want to do it again, I really don’t but it’s, you know, depression when it comes, it’s the only thing I can turn to.”

Physical symptoms were common in the 12 months preceding sexual assault and reported more frequently afterwards with a near doubling in experience of poor sleep (47% vs. 87%) and a three-fold increase in change in appetite (27% vs. 75%). Qualitative data highlighted the substantial impact of the assault on sleep problems and their pervasive influence on other outcomes. Nightmares, insomnia and lack of a sleep routine were attributed to anxiety, panic attacks and flashbacks, and were exacerbated by more general worries over school, exams, foster placements and the police investigation. Sleep deprivation and disturbance in turn were held responsible for heightened anxiety and panic attacks, migraines, poor concentration, mood swings and irritability and, indirectly, for disinclination to attend school and work and inability to sustain friendships.

“Like today when I woke up I said I couldn’t go to college, […] I didn’t want to get up, I feel so depressed, and I’m just like I can’t go on […] I don’t want to talk to no-one, I just want to be by myself […]”

Two thirds of participants had visited a health service for a physical health problem in the 12 months prior to the assault, compared to four fifths between assault and study end (65% vs 82%). Similarly, nearly half accessed mental health help in the 12 months prior, and four fifths between the assault and study end (47% vs. 80%). Changes were seen in the number of participants accessing support from a mental health professional (29% vs. 60%), a counsellor (27% vs. 42%) or the voluntary sector or alternative therapies (3% vs. 18%).

### Social outcomes

Table 4 shows social outcomes following assault for all participants. Female-only data can be found in Supplementary Material. Fourteen percent (14%) of participants had experienced re-victimisation by study end and the proportion ever in foster care had nearly doubled (18% vs. 32%). Persistent absence from school doubled (22% vs. 47%) over the course of the study. Rates of being NEET were high throughout but reduced from 28 to 15%. Three of 22 participants aged < 16 years at study end were not in school (14%). Overall, those disengaged from education and employment rose from 31 to 41%.

**Table 4:** Social outcomes following sexual assault

	All participants followed up to study end (n = 75)				
	*N*^a^	Study entry	Study end	*P* value*	% diff.
		*n* (%)	*n* (%)		
Re-victimisation by study end (cumulative)	73	–	10 (13.7%)	–	–
Ever in foster care					
Prior to or at the time of the assault	74	13 (17.6%)	–	–	–
By study end (cumulative)	72	–	23 (31.9%)	–	–
Education and employment					
All ages: in education or employment	75	66 (88.0%)	64 (85.3%)	0.815	2.7%
All ages: missed > 30 days of school in last 12 months^b^	51	11 (21.6%)	24 (47.1%)	**0.004**	− 25.5%
Participants aged 13–15 years at study entry who were not in school	75	0	–	–	–
Participants aged 13–15 years at study end who were not in school	22	–	3 (13.6%)	–	–
Participants aged 16 years + at study entry who were not in education, employment or training (NEET)	32	9 (28.1%)	5 (15.6%)	0.388	12.5%
Participants aged 16 years + at study end who were not in education, employment or training (NEET)	53	–	8 (15.1%)	–	–
All ages: disengaged from education and employment^c^	75	23 (30.7%)	31 (41.3%)	0.169	− 10.7%

*McNemar Test: *P* values < 0.05 were considered significant and are in bold

^a^*N* = number with valid data

^b^Denominator is those in school

^c^All those who were (i) in school but had missed > 30 days of school in last 12 months or (ii) not in school (13–15y) or NEET (16y +)

Qualitative data illuminated the mechanisms at work in the strong association between assault and persistent absence from school. Mental health issues impacted on attendance, concentration, and performance. Sleep problems led to difficulty getting up and out, and some were immobilised and house-bound by panic attacks and agoraphobia. Absence for court hearings and other appointments disrupted attendance and, where the assailant was a fellow pupil, the young person was disinclined to attend school alongside them or their peers (for fear of repercussions). Once at school or college, lethargy, lack of motivation and inability to concentrate, compounded by feelings of worthlessness and low self-esteem, hindered educational progress and attainment.

“For a while I just couldn’t do it [school], I couldn’t, I didn’t want to be around people, I just wanted to be alone. I even used to find it hard to get out of my bed, I just wanted to be in the dark, zoned out […]”

“[…] like usually when I go to court […] I can’t go to school the next day because I’ll be a bit upset and like I just can’t be bothered to see anyone […]”

The qualitative data also suggested a bidirectional relationship between mental health problems and disengagement from education. Anxiety and depression had a detrimental effect on academic performance which in turn led to further mental health and sleep problems resulting in a vicious spiral. Deterioration in conduct, including outbursts of anger and aggression, caused disciplinary issues, particularly where teachers were unaware of the assault. Participants reported a lack of understanding on the part of schools of how to support them post-assault and few allowances having been made for its effects on academic performance. Some were excluded, others dropped out permanently or temporarily, or repeated a year, and several were relocated to another school.

“ [...] so I didn’t go to school for a long time, then going back so then I was behind and I’m not coming in some days because I just feel like I just don’t want to see anyone and then into class I wouldn’t concentrate like it feels just all really long so my school was just like, your attendance is really bad, like you’re not going to get good [grades] [...] so it was a thing where it was best me just to drop out and just, yeah.”

Educational attainment was adversely affected with lasting consequences for career aspirations.

“I was looking at [xxxx] University for example to do medicine, you need five A*, I got two, I was predicted eight, so if that never happened, I could have gone to [xxxx].”

What quantitative data were unable to capture, and in-depth accounts illustrated vividly, was the impact of the assault on relationships with family members, partners and peers and their importance for outcomes. Initial disruptions to close relationships were common. Most participants described increased tension at home in the months following assault. Parents’ first reaction was often to be over-protective, curtailing their child’s freedom and causing them to feel untrusted and punished. Participants felt guilt at having upset their parents. Those in sexual relationships reported increased arguments and difficulties engaging in sexual activity. One participant whose boyfriend assaulted her grieved the loss of this relationship. Friendships deteriorated or ended due to a combination of social withdrawal and uncertainty about how to manage the relationship.

“[…] people would say rape jokes just to get a reaction out of me and that just made my temper even worse […]”

“… after it happened, they didn’t really know how to treat me. They didn’t know if I needed the support or if I just wanted to forget about it and because I never brought it up, they just never brought it up but they’d always kind of tread on eggshells.”

Longer-term outcomes however were often more positive. Relationships with some parents became closer following emotional disclosure; new, healthier friendships were forged; and relationships with teachers improved, especially if the young person changed schools and had a fresh start. In these instances, friends and family were seen as being important in providing beneficial support and reassurance.

“[…] there was a girl at school actually [...] she was the one who, like, helped me through, didn’t judge me, I think she was like the first person who made me laugh.[...] she didn’t ask too much questions like everyone else did, about what happened, [...] the only thing she asked me was, okay, do you want to go and do this?”

“[Mum and I] were having arguments about the fact that I wasn’t opening up to her. Because I have been known to bottle things up a lot, but yeah, after all of it came out [...] I wasn’t always in bed, like just being on my own. I was actually, like, playing with the dog and going out in the garden with her, [...] spending time with people for once.”

### Associations with key outcomes

Tables 5, 6 shows associations with key medium-term outcomes including mental health symptoms above threshold and disengagement from education and employment, for females only. A sensitivity analysis was conducted to include all participants and the results were similar with no major differences.

**Table 5:** Crude and adjusted associations between socio-demographic and assault characteristics, vulnerability factors and service use, and adverse mental health outcomes among female participants only

Mental health symptom questionnaire score above threshold at study end (females)																		
	CRIES^a^						SCARED^b^						SMFQ^c^					
	*N*	n/N (%) with outcome	Crude OR	*p*	AOR^d^ (95% CI)	*p*	*N*	*n*/*N* (%) with outcome	Crude OR	*p*	AOR^d^ (95% CI)	*p*	*N*	*n*/*N* (%) with outcome	Crude OR	*p*	AOR^d^ (95% CI)	*P*
Demographic factors																		
Age			0.98	0.900					0.69	0.055					1.03	0.859		
Ethnicity																		
White	32	26 (81%)	1.00				32	17 (53%)	1.00				32	17 (53%)	1.00			
Mixed	9	4 (44%)	0.18	**0.037**			9	5 (56%)	1.10	0.897			10	4 (40%)	0.59	0.471		
Asian	1	1 (100%)	1*				1	0	1*				1	0	1*			
Black	25	18 (72%)	0.59	0.411			24	18 (75%)	2.65	0.099			25	16 (64%)	1.57	0.410		
Other	2	1 (50%)	0.23	0.323			2	1 (50%)	0.88	0.932			2	1 (50%)	0.88	0.932		
Index of multiple deprivation																		
Quintile 1 (most deprived)	18	15 (83%)	1.00				19	12 (63%)	1.00				19	12 (63%)	1.00			
Quintile 2	28	20 (71%)	0.50	0.361			26	13 (50%)	0.58	0.382			28	12 (43%)	0.44	0.175		
Quintiles 3–5 (least deprived)	23	15 (65%)	0.38	0.202			23	16 (70%)	1.33	0.661			23	14 (61%)	0.91	0.879		
Assault characteristics																		
Violent assault: physical violence or weapons	37	30 (81%)	1.88	0.309	2.30 (0.60, 8.83)	0.226	36	23 (64%)	1.36	0.572	2.00 (0.55, 7.32)	0.294	37	24 (65%)	2.18	0.145	2.53 (0.78, 8.25)	0.123
Multiple assailants	15	11 (33%)	1.09	0.901	1.21 (0.30, 3.95)	0.793	14	10 (71%)	1.92	0.319	1.75 (0.45, 6.88)	0.421	15	9 (60%)	1.39	0.576	1.35 (0.39, 4.66)	0.634
Substance use around assault	20	16 (80%)	1.60	0.473	3.94 (0.79, 19.62)	0.094	19	11 (58%)	0.76	0.634	1.73 (0.40, 7.43)	0.463	20	11 (55%)	0.83	0.736	1.12 (0.32, 3.96)	0.863
Stranger assault	25	18 (72%)	0.83	0.745	0.85 (0.25, 2.85)	0.786	25	14 (56%)	0.81	0.690	0.70 (0.22, 2.20)	0.540	25	13 (52%)	0.81	0.682	0.77 (0.26, 2.26)	0.631
Vulnerability factors																		
Foster care pre- or at time of assault	12	7 (58%)	0.42	0.196	1.26 (0.21, 7.72)	0.802	10	6 (60%)	0.94	0.933	1.44 (0.25, 8.51)	0.685	12	7 (58%)	1.17	0.803	2.98 (0.54, 16.58)	0.212
Ever in foster care by study end	20	13 (65%)	0.66	0.464	1.25 (0.31, 5.00)	0.764	18	13 (72%)	2.29	0.169	3.00 (0.67, 13.46)	0.152	20	13 (65%)	1.94	0.231	3.14 (0.81, 12.13)	0.097
Social services pre- or at time of assault	24	19 (79%)	1.65	0.408	4.43 (0.85, 22.99)	0.076	22	17 (77%)	3.25	**0.047**	6.07 (1.34, 27.63)	**0.020**	24	20 (83%)	7.65	**0.001**	28.22 (3.35, 238.05)	**0.002**
Special educational needs statement	12	8 (67%)	0.70	0.603	0.54 (0.11, 2.71)	0.451	12	8 (67%)	1.48	0.557	2.66 (0.58, 12.11)	0.206	12	8 (67%)	1.93	0.326	2.60 (0.61, 11.14)	0.197
Self-harm in 12 months pre-assault	26	20 (80%)	1.33	0.618	1.40 (0.41, 4.72)	0.591	25	16 (64%)	1.33	0.580	1.47 (0.47, 4.54)	0.507	26	18 (69%)	2.59	0.069	2.43 (0.83, 7.10)	0.104
Prior history of sexual abuse/assault	23	17 (74%)	1.15	0.808	1.17 (0.34, 4.03)	0.799	22	15 (68%)	1.57	0.413	1.44 (0.44, 4.65)	0.545	23	13 (57%)	1.09	0.864	0.92 (0.31, 2.70)	0.878
Re-victimisation by study end	10	9 (90%)	3.83	0.220	4.24 (0.45, 40.32)	0.208	9	8 (89%)	6.71	0.082	7.28 (0.75, 70.82)	0.087	10	8 (80%)	4.00	0.096	3.63 (0.66, 20.03)	0.139
Mental health																		
Mental health help in 12 months pre-assault	32	25 (78%)	1.71	0.330	2.62 (0.72, 9.60)	0.145	31	21 (68%)	1.79	0.253	2.03 (0.66, 6.30)	0.218	32	20 (63%)	1.85	0.207	2.25 (0.77, 6.59)	0.140
Mental health disorder at first follow-up	41	35 (85%)	5.83	**0.022**	5.35 (1.09, 26.14)	**0.038**	39	25 (64%)	2.68	0.175	3.84 (0.71, 20.67)	0.117	41	26 (63%)	4.04	0.067	5.70 (1.00, 32.32)	**0.049**
Mental health help accessed post-assault	55	42 (76%)	2.77	0.112	2.31 (0.57, 9.43)	0.244	53	32 (60%)	1.31	0.668	1.13 (0.28, 4.56)	0.87	55	30(55%)	1.03	0.964	0.85 (0.22, 3.25)	0.808

*P* values < 0.05 were considered significant and are in bold

^*^Empty

^a^Child revised impact of events scale

^b^Screen for child anxiety-related disorder

^c^Short moods and feelings questionnaire

^d^Adjusted odds ratio; adjusted for ethnicity, deprivation and age

**Table 6:** Crude and adjusted associations between socio-demographic and assault characteristics, vulnerability factors and service use, and an adverse education/employment outcome among female participants only

Disengaged from education/employment at study end (females)						
	*N*	*n*/*N* (%) with outcome	Crude OR	*P*	AOR^a^ (95% CI)	*p*
Demographic factors						
Age			0.80	0.222		
Ethnicity						
White	32	16 (50%)				
Mixed	10	4 (40%)	0.67	0.582		
Asian	1	0	1*			
Black	26	8 (31%)	0.44	0.142		
Other	2	2 (100%)	1*			
Index of multiple deprivation						
Quintile 1 (most deprived)	19	7 (37%)	1.00			
Quintile 2	28	8 (29%)	0.69	0.551		
Quintiles 3–5 (least deprived)	24	15 (63%)	2.86	0.099		
Assault characteristics						
Violent assault: physical violence or weapons	37	18 (49%)	1.21	0.719	1.14 (0.34, 3.78)	0.830
Multiple assailants	16	6 (38%)	0.75	0.623	1.00 (0.28, 3.63)	0.995
Substance use around assault	20	10 (50%)	1.26	0.667	0.98 (0.24, 3.96)	0.978
Stranger assault	25	9 (36%)	0.65	0.399	0.35 (0.10, 1.31)	0.120
Vulnerability factors						
Foster care pre- or at time of assault	12	8 (67%)	3.27	0.077	11.23 (1.57, 80.28)	**0.016**
Ever in foster care by study end	21	13 (62%)	3.15	**0.035**	5.41 (1.28, 22.76)	**0.021**
Social services pre- or at time of assault	25	17 (68%)	5.49	**0.002**	10.57 (2.58, 43.38)	**0.001**
Special educational needs statement	12	7 (58%)	2.33	0.191	2.14 (0.46, 10.02)	0.332
Self-harm in 12 months pre-assault	27	14 (52%)	2.01	0.163	1.93 (0.63, 5.93)	0.248
Prior history of sexual abuse/assault	24	10 (42%)	0.93	0.884	1.04 (0.33, 3.26)	0.940
Re-victimisation by study end	10	7 (70%)	3.92	0.065	3.74 (0.79, 17.66)	0.096
Mental health						
Mental health help in 12 months pre-assault	33	19 (58%)	3.33	**0.017**	2.50 (0.82, 7.64)	0.109
Mental health disorder at first follow-up	41	21 (51%)	9.45	**0.041**	8.96 (0.88, 91.20)	0.064
Mental health help accessed post-assault	56	24 (43%)	0.88	0.829	0.48 (0.10, 2.17)	0.337

*P* values < 0.05 were considered significant and are in bold

^a^Adjusted odds ratio; adjusted for ethnicity, deprivation and age

*Empty

Univariate regression analyses showed an association between enduring PTS symptoms and mixed ethnicity. Social services involvement prior to or at the time of assault predicted enduring anxiety and depressive symptoms at study end, including after adjusting for age, ethnicity and deprivation. Mental health disorder at first (4–5 month) follow-up predicted enduring PTS and depressive symptoms at study end after adjusting for the same factors.

Social services involvement prior to or at the time of assault, mental health help in the 12 months pre-assault and mental health disorder at first follow-up were also associated with disengagement from education and employment at study end, although only social care involvement remained significant after adjusting for age, ethnicity and deprivation. There was an association between being in foster care at study entry or end and an adverse education/employment outcome.

No associations were observed between assault characteristics and mental health or education/employment outcomes.

## Discussion

### Summary of key findings

This vulnerable cohort of adolescents followed-up after serious sexual assault had improved mental health symptom outcomes 13–15 months post-assault but levels of morbidity and self-harm remained very high and social outcomes were poor. A quarter reported new onset self-harm and participants were more likely to report physical symptoms of poor sleep and appetite change following the assault. Four in five participants accessed mental health help in the interval after sexual assault reflecting the cohort’s level of need. Key factors associated with adverse mental health symptom outcomes included social care involvement prior to the assault and presence of mental health disorder 4–5 months post-assault. Previous social care involvement and ever experience of foster care were associated with disengagement from education or employment following assault.

Qualitative data amplified the quantitative findings, providing insights into what the assault meant for participants’ quality of life and perceived life chances, and for their mental and physical health. What emerged clearly is the interrelationship between many of the outcomes: mental health problems, sleep deprivation and disturbance, damage to relationships and diminished life chances, such that harms were cumulative. The qualitative data also illustrated the bi-directionality in associations between outcomes. The adverse mental health impact of the assault led to poorer functioning and performance, which in turn increased mental ill-health.

### Comparison with population norms

Physical complaints, such as sleep difficulties, which are reported by around a third of young people generally [38–40], were higher than population estimates. Cohort rates of mental health symptoms, at the level indicating ‘risk for’ disorder, remained substantially elevated when compared with population rates of mental health disorder (14% and 17% among 11–16 and 17–19 year olds in England, respectively) [41]. Prevalence of self-harm was very high pre-and post-assault relative to population rates of 26% among 11–16 year olds with a mental health disorder and 3% of those without [41]. Smoking rates over the course of the study were at least three-fold higher than comparable population prevalence [42], whereas increases in ever drug use and rates of drinking were comparable with population estimates [42, 43]. Binge drinking or getting drunk in the last month, also comparable with population estimates, remained stable, although individual trajectories differed following experience of assault.

The very poor social outcomes for participants contrast greatly with general population levels: persistent school absence in 47% compared with 5.4% of English secondary school children during the 2014–15 school year (15.4% among pupils attending special schools) [44]. 15% of participants were NEET at study end relative to 3–5% nationally [45].

### Comparison with literature

In common with a small number of other longitudinal studies following-up young people after sexual assault [16], mental health symptoms in this study declined over time. However, the majority of participants’ scores remained above thresholds indicating risk for disorder, signalling ongoing clinical need. The present study’s results mirror previous studies’ findings for PTS symptoms but present stronger evidence for ongoing depressive and anxiety symptoms. The high prevalence of self-harm indicates high levels of distress and could reflect presence of depressive disorder, emotion dysregulation, patterns of maladaptive coping or relationship difficulties [46, 47]. Emotion dysregulation was particularly associated with impairment and further exposure to trauma in the first months after sexual assault [47]. Lifetime social services involvement was a predictor of persisting mental health morbidity at study end, demonstrating clustering of risks and adverse outcomes. There is little comparative literature in this area, and conclusions drawn from studies of various trauma [48] or sexual assault in older populations [49] have been mixed.

Few studies exist regarding sexual assault sequelae relating to education [50], although longitudinal associations between mental health disorder and poor educational outcomes have been described previously [51, 52]. Several studies have reported cross-sectional associations between adolescent sexual assault and poorer educational outcomes [3, 11, 14]**,** including high school drop-out [53]. Qualitatively, Nikischer [54] illustrated how some US survivors of adolescent sexual violence feel that these experiences derailed their education with lasting impact. Despite the well-known association between being in foster care and adverse educational outcomes [55], there appear to be no studies evaluating outcomes for this group following sexual assault. Foster care may have a positive impact on school attendance [56], suggesting other factors may influence disengagement from school or employment following sexual assault.

### Strengths and limitations

This prospective, longitudinal study achieved high retention of a sizeable, representative sample from an adolescent population traditionally thought difficult to recruit and retain. The specific focus on adolescents distinguishes this study from much of the literature, which tends to focus on adult populations of sexual assault. The mixed methods design, with qualitative descriptions enhancing the quantitative data findings, and longitudinal nature help elucidate some of the risk factors for worse mental health and educational outcomes among those experiencing sexual assault whilst minimising recall bias. Generalizability may be limited due to recruitment from those attending a sexual assault service. It is unknown whether vulnerability and complexity may have been over- or under-represented in this cohort. Data from the SCARED, S-MFQ and CRIES questionnaires on anxiety, depression, and post-traumatic stress symptoms are likely to be more reliable than data from the SDQ on behavioural symptoms, because it was not possible in most cases to include a second informant [26]. Applicability is high as a wrap-around suite of measures of function were made. However, evaluation of social outcomes was limited by a lack of measures of assault impact on relationships. The impact of experience of foster care on educational outcome was not explored in the qualitative interviews. Other limitations affecting precision include: the overall small sample size affecting power; under-representation of South Asians and males; and absence of a comparable control group limiting assessment of causality. Education and employment outcomes reported by young people were not independently verified.

### Recommendations for services/policy makers

This study demonstrated a high level of need among adolescents 13–15 months post-sexual assault. Participants had multiple disadvantage at baseline, suggesting a relationship between clustering of risk and complex co-morbidities post assault, signalling the need for practitioners to be alert and respond to the likely needs of adolescents with a wider risk profile. The likely inter-dependence of factors affecting outcome supports the need for multi-modal, multi-agency packages of support that also address educational needs, re-victimisation risk and the particular challenges for looked-after young people.

The marked effect of assault on engagement with education, the strong plea by young people for support from schools in helping them remain in education, and schools’ apparent lack of preparedness to do so, has increasingly been recognised [57]. Gaps relating to sexual violence in previous guidance for schools [58] have been addressed in subsequent policy documents [59] but more needs to be done in terms of enactment.

### Recommendations for research

This work would benefit from replication in a larger sample that is also able to evaluate the needs of adolescent boys following assault.

Understanding the mediating factors in the associations between sexual assault and its adverse outcomes is important in informing intervention strategies to mitigate the harms. On the evidence from young people, their challenges post sexual assault were neither optimally understood nor adequately acknowledged in educational settings, calling for interventional research.

Personal relationships with friends and family members, by contrast, emerged as valuable sources of support for some. Past research has highlighted the role of social relationships in providing support in the context of sexual assault and other stressful experiences [60–63]. The evidence in this study for the importance, positive or negative, of relationships with friends and family warrants further empirical investigation and makes the case for including measures of the quality of such relationships in data routinely collected in the service setting and for carrying out further research to determine the role of social and personal relationships in increasing resilience.

In conclusion, multi-faceted support is needed to prevent social exclusion and further widening of health inequalities in this population, promote recovery following sexual assault and enable young people to lead fuller lives.

### Supplementary Information

Below is the link to the electronic supplementary material.

Supplementary file1 (DOCX 29 kb)

## Data Availability

Full Tables with information for all participants and females only are available. SK is willing to discuss data sharing on request.

## References

[1] WHO DoRHaR, London School of Hygiene and Tropical Medicine, South African Medical Research Council (2013). Global and regional estimates of violence against women: prevalence and health effects of intimate partner violence and non-partner sexual violence.

[2] Macdowall W, Gibson LJ, Tanton C, Mercer CH, Lewis R, Clifton S, Field N, Datta J, Mitchell KR, Sonnenberg P, Erens B, Copas AJ, Phelps A, Prah P, Johnson AM, Wellings K (2013). Lifetime prevalence, associated factors, and circumstances of non-volitional sex in women and men in Britain: findings from the third National Survey of Sexual Attitudes and Lifestyles (Natsal-3). Lancet.

[3] UNICEF (2012) Progress for children: a report card on adolescents. No. 10, April 2012. United Nations Children's Fund, New York

[4] Barter CMM, Berridge D (2009). (2009) Partner exploitation and violence in teenage intimate relationships.

[5] Halpern CT, Young ML, Waller MW, Martin SL, Kupper LL (2004). Prevalence of partner violence in same-sex romantic and sexual relationships in a national sample of adolescents. J Adolesc Health.

[6] Khadr SN, Viner RM, Goddard A (2011). Safeguarding in adolescence: under-recognised and poorly addressed. Arch Dis Child.

[7] Wood M, Barter C, Berridge (2011) Standing on my own two feet: disadvantaged Teenagers, Intimate Partner Violence and Coercive Control. (London:NSPCC 2011) https://www.nspcc.org.uk/globalassets/documents/research-reports/standing-own-two-feet-report.pdf. Accessed 22 July 2021

[8] Mokdad AH, Forouzanfar MH, Daoud F (2016). Global burden of diseases, injuries, and risk factors for young people’s health during 1990–2013: a systematic analysis for the Global Burden of Disease Study 2013. Lancet.

[9] WHO (2017) Responding to children and adolescents who have been sexually abused. WHO clinical guidelines. World Health Organization, Geneva. http://apps.who.int/iris/bitstream/handle/10665/259270/9789241550147-eng.pdf;sequence=1. Accessed 22 July 2021 29630189

[10] Silverman JG, Raj A, Mucci LA, Hathaway JE (2011). Dating violence against adolescent girls and associated substance use, unhealthy weight control, sexual risk behaviour, pregnancy and suicidality. JAMA.

[11] Martz DM, Jameson JP, Page AD (2016). Psychological health and academic success in rural Appalachian adolescents exposed to physical and sexual interpersonal violence. Am J Orthopsychiatry.

[12] Kilpatrick DG, Ruggiero KJ, Acierno R, Saunders BE, Resnick HS, Best CL (2003). Violence and risk of PTSD, major depression, substance abuse/dependence, and comorbidity: results from the national survey of adolescents. J Consult Clin Psychol.

[13] Decker MR, Peitzmeier S, Olumide A (2014). Prevalence and health impact of intimate partner violence and non-partner sexual violence among female adolescents aged 15–19 years in vulnerable urban environments: A multi-country study. J Adolesc Health.

[14] Banyard VL, Cross C (2008). Consequences of teen dating violence: understanding intervening variables in ecological context. Violence Against Women.

[15] Khadr S, Clarke V, Wellings K, Villalta L, Goddard A, Welch J, Bewley S, Kramer T, Viner R (2018). Mental and sexual health outcomes following sexual assault in adolescents: a prospective cohort study. Lancet Child Adolesc Health.

[16] MacGregor KE, Villalta L, Clarke V, Viner R, Kramer T, Khadr SN (2019). A systematic review of short and medium-term mental health outcomes in young people following sexual assault. J Child Adolesc Ment Health.

[17] Ackard DM, Neumark-Sztainer D (2002). Date violence and date rape among adolescents: associations with disordered eating behaviors and psychological health. Child Abuse Negl.

[18] Burgess AW, Hartman CR, Clements PT (1995). Biology of memory and childhood trauma. J Psychosoc Nurs Ment Health Serv.

[19] Putnam FW (2003). Ten-year research update review: child sexual abuse. J Am Acad Child Adolesc Psychiatry.

[20] Burnett S, Bird G, Moll J, Frith C, Blakemore SJ (2009). Development during adolescence of the neural processing of social emotion. J Cogn Neurosci.

[21] Mills KL, Goddings AL, Clasen LS, Giedd JN, Blakemore SJ (2014). The developmental mismatch in structural brain maturation during adolescence. Dev Neurosci.

[22] Haavet OR, Sagatun Å, Lien L (2011). Adolescents’ adverse experiences and mental health in a prospective perspective. Scand J Pub Health.

[23] National Institute for Clinical Excellence (2005) Post-traumatic stress disorder: the management of PTSD in adults and children in primary and secondary care. Gaskell and the British Psychological Society, London. https://www.nice.org.uk/guidance/cg26/evidence/full-guideline-including-appendices-113-pdf-193442221. Accessed 22 July 2021

[24] Pine DS, Cohen E, Cohen P (1999). Adolescent depressive symptoms as predictors of adult depression: moodiness or mood disorder?. Am J Psychiatry.

[25] Payne RA, Abel GA (2012). UK indices of multiple deprivation–a way to make comparisons across constituent countries easier. Health Stat Q.

[26] Goodman R, Ford T, Richards H, Gatward R, Meltzer H (2000). The development and well-being assessment: description and initial validation of an integrated assessment of child and adolescent psychopathology. J Child Psychol Psychiatry.

[27] Perrin S, Meiser-Stedman R, Smith P (2005). The children’s revised impact of event scale (CRIES): validity as a screening instrument for PTSD. Behav Cogn Psychother.

[28] Angold A, Costello EJ, Messer SC, Pickles A (1995). Development of a short questionnaire for use in epidemiological studies of depression in children and adolescents. Int J Methods Psychiatr Res.

[29] Birmaher B, Brent DA, Chiappetta L, Bridge J, Monga S, Baugher M (1999). Psychometric properties of the screen for child anxiety related emotional disorders (SCARED): a replication study. J Am Acad Child Adolesc Psychiatry.

[30] NatCen Social Research (2011) Smoking, drinking and drug use among young people in England in 2010. UK Data Service. http://content.digital.nhs.uk/catalogue/PUB00396/smok-drin-drug-youn-peop-eng-2010-rep2.pdf. Accessed 22 July 2021

[31] NHS National Services Scotland. Information Services Division (2013) Scottish schools adolescent lifestyle and substance use survey, 2010. UK Data Service. SN: 7375. 10.5255/UKDA-SN-7375-1, https://www.isdscotland.org/Health-Topics/Public-Health/Publications/2014-11-25/SALSUS_2013_National_Overview.pdf. Accessed 22 July 2021

[32] Hibell B, Guttormsson U, Ahlström S et al (2012) The 2011 ESPAD report: substance use among students in 36 European countries. Swedish Council for Information on Alcohol and Other Drugs, Stockholm. http://espad.org/sites/espad.org/files/The_2011_ESPAD_Report_FULL_2012_10_29.pdf. Accessed 22 July 2021

[33] Youth In Mind (2006) Scoring the SDQ: Syntax for SPSS. http://www.sdqinfo.org/c1.html. Accessed 22 July 2021

[34] Youth In Mind (2010) Scoring the SDQ: Syntax for STATA. http://www.sdqinfo.org/c3.html. Accessed 22 July 2021

[35] Ritchie J, Lewis J (2003). Qualitative research practice: a guide for social science students and researchers.

[36] Silverman D (2010). Doing qualitative research: a practical handbook.

[37] Brannen J (2005). Mixing methods: the entry of qualitative and quantitative approaches into the research process. Int J Soc Res Methodol Spec Issue.

[38] Ohayon MM, Roberts RE, Zulley J, Smirne S, Priest RG (2000). Prevalence and patterns of problematic sleep among older adolescents. J Am Acad Child Adolesc Psychiatry.

[39] Viner R, Christie D, Viner R (2005). Fatigue and chronic symptoms. ABC of adolescence.

[40] Inchley J et al (eds) (2016) Growing up unequal: gender and socioeconomic differences in young people's health and well-being. Health Behaviour in School-aged Children (HBSC) study: international report from the 2013/2014 survey. Copenhagen, WHO Regional Office for Europe (Health Policy for Children and Adolescents, No. 7). https://www.euro.who.int/__data/assets/pdf_file/0003/303438/HSBC-No.7-Growing-up-unequal-Full-Report.pdf. Accessed 22 July 2021

[41] NHS Digital (2018) Mental health of children and young people in England 2017. https://digital.nhs.uk/data-and-information/publications/statistical/mental-health-of-children-and-young-people-in-england/2017/2017. Accessed 22 July 2021

[42] NHS Digital (2019) Smoking, drinking and drug use among young people in England 2018 [NS]. https://digital.nhs.uk/data-and-information/publications/statistical/smoking-drinking-and-drug-use-among-young-people-in-england/2018. Accessed 22 July 2021

[43] Office for National Statistics (2013) Opinions and Lifestyle Survey. http://www.ons.gov.uk/ons/about-ons/products-and-services/opn/index.html. Accessed 22 July 2021

[44] Department for Education (2016) Pupil absence in schools in England: 2014–2015. England. https://www.gov.uk/government/uploads/system/uploads/attachment_data/file/509989/SFR10_2016_text.pdf. Accessed 22 July 2021

[45] Department for Education (2017). Participation in education, training and employment by 16–18 year olds in England. England. https://www.gov.uk/government/uploads/system/uploads/attachment_data/file/623310/SFR29_2017_Main_text_.pdf. Accessed 22 July 2021

[46] Valencia-Agudo F, Kramer T, Clarke V, Goddard A, Khadr S (2020). Correlates and predictors of self-harm in a prospective sample of sexually assaulted adolescents. Clin Child Psychol Psychiatry.

[47] Villalta L, Khadr S, Chua K-C, Kramer T, Clarke V, Viner RM, Stringaris A, Smith P (2020). Complex post-traumatic stress symptoms in female adolescents: the role of emotion dysregulation in impairment and trauma exposure after an acute sexual assault. Eur J Psychotraumatol.

[48] Trickey D, Siddaway AP, Meiser-Stedman R, Serpell L, Field AP (2012). A meta-analysis of risk factors for post-traumatic stress disorder in children and adolescents. Clin Psychol Rev.

[49] Darves-Bornoz JM, Lepine JP, Choquet M, Berger C, Degiovanni A, Gaillard P (1998). Predictive factors of chronic post-traumatic stress disorder in rape victims. Eur Psychiatry.

[50] Baker MR, Frazier PA, Greer C (2016). Sexual victimization history predicts academic performance in college women. J Couns Psychol.

[51] Fergusson DM, Woodward LJ (2002). Mental health, educational, and social role outcomes of adolescents with depression. Arch Gen Psychiatry.

[52] Woodward LJ, Fergusson DM (2001). Life course outcomes of young people with anxiety disorders in adolescence. J Am Acad Child Adolesc Psychiatry.

[53] Timothy M, Diette AHG, Hamilton D, Darity Jr WA (2017). Child abuse, sexual assault, community violence and high school graduation. Rev Behav Econom.

[54] Nikischer AB (2014) Dreams deferred: e impact of sexual assault during adolescence on the educational outcomes and life choices of women. Adult Edu Res Conf. http://newprairiepress.org/aerc/2014/papers/58. Accessed 22 July 2021

[55] Department for Education (2017) Outcomes for children looked-after by LAs: 31 March 2016: SFR12/2017. Department for Education, London. https://assets.publishing.service.gov.uk/government/uploads/system/uploads/attachment_data/file/602087/SFR12_2017_Text.pdf. Accessed 22 July 2021

[56] Department for Education (2019) Outcomes for children looked after by local authorities in England, 31 March 2018. https://assets.publishing.service.gov.uk/government/uploads/system/uploads/attachment_data/file/794535/Main_Text_Outcomes_for_CLA_by_LAs_2018.pdf. Accessed 22 July 2021

[57] House of Commons (2016) “Sexual harassment and sexual violence in schools” noted “Schools lack the guidance, training and structures to deal with incidents of sexual harassment and sexual violence”. UK. https://publications.parliament.uk/pa/cm201617/cmselect/cmwomeq/91/9106.htm. Accessed 22 July 2021

[58] Department for Education (2015) Keeping children safe in education: statutory guidance for schools and colleges. https://www.gov.uk/government/publications/keeping-children-safe-in-education--2. Accessed 22 July 2021

[59] Department for Education (2017) Sexual violence and sexual harassment between children in schools and colleges: advice for governing bodies, proprietors, headteachers, principals, senior leadership teams and designated safeguarding leads. https://www.gov.uk/government/publications/sexual-violence-and-sexual-harassment-between-children-in-schools-and-colleges. Accessed 22 July 2021

[60] Dworkin ER, Menon SV, Bystrynski J, Allen NE (2017). Sexual assault victimization and psychopathology: a review and meta-analysis. Clin Psychol Rev.

[61] Fergerson AK, Brausch AM (2020). Resilience mediates the relationship between PTSD symptoms and disordered eating in college women who have experienced sexual victimization. J Interpers Viol.

[62] Haden SC, Scarpa A, Jones RT, Ollendick TH (2007). Posttraumatic stress disorder symptoms and injury: the moderating role of perceived social support and coping for young adults. Personality Individ Differ.

[63] Livingston JA, Hequembourg A, Testa M, VanZile-Tamsen C (2007). Unique aspects of adolescent sexual victimization experiences. Psychol Women Q.

