# Supplementary material for: Medium-term health and social outcomes in adolescents following sexual assault: a prospective mixed-methods cohort study
Source: Soc Psychiatry Psychiatr Epidemiol. 2021 Aug 9;58(12):1777–93. doi: 10.1007/s00127-021-02127-4 (PMC10627884; doi:10.1007/s00127-021-02127-4)
Supplement: Supplementary file 1 — Supplementary file1 (DOCX 29 kb) [file 127_2021_2127_MOESM1_ESM.docx]

**Supplementary Material**

**Table A: Longitudinal changes in mental health symptoms and substance use among female participants**

|  |  | **Female participants followed-up to study end (N=71)** | | | | |
| --- | --- | --- | --- | --- | --- | --- |
|  |  |  | **Study entry** | **Study end** |  |  |
|  |  | N^e^ | n (%) | n(%) | P value* | % diff |
| **Mental Health Symptom score ≥ threshold** |  |  |  |  |  |  |
| CRIES -13 ^b^ |  | 67 | 61 (91.0%) | 49 (73.1%) | **0.008** | 17.9% |
| S-MFQ ^c^ |  | 68 | 61 (89.7%) | 37 (54.4%) | **<0.000** | 35.3% |
| SCARED ^d^ |  | 63 | 48 (76.2%) | 38 (60.3%) | **0.031** | 15.9% |
| SDQ: Total Score ^e^ |  | 66 | 24 (36.4%) | 16 (24.2%) | 0.057 | 12.1% |
|  |  |  |  |  |  |  |
| **Substance use** |  |  |  |  |  |  |
| Current smoker |  | 68 | 24 (35.3%) | 29 (42.6%) | 0.302 | -7.4% |
| Current alcohol use |  | 69 | 42 (60.9%) | 54 (78.3%) | **0.004** | -17.4% |
| Binge drinking in last month |  | 63 | 15 (23.8%) | 16 (25.4%) | 1.000 | -1.6% |
| Drunk in the last month |  | 65 | 15 (23.1%) | 17 (26.2%) | 0.804 | -3.1% |
| Ever used drugs (at study entry) |  | 70 | 28 (40.0%) | - | - | - |
| Ever used drugs by study end (cumulative measure) |  | 65 | - | 38 (58.5%) | - | - |

^a^ N = number with valid data

^b^ Child Revised Impact of Events Scale; cut-off score of ≥30;

^c^ Short Moods and Feelings Questionnaire; cut-off score of ≥8;

^d^ Screen for Child Anxiety Related Disorder; cut-off score of ≥30, cases with ≥30% missing data excluded;

^e^ Strengths and Difficulties Questionnaire; cut-off score of ≥20

***McNemar Test. P-values <0.05 were considered significant and are in bold

**Table B: Changes in self-harm, physical symptoms and health service use following sexual assault among female participants**

|  |  | **Female participants followed-up to study end (N=71)** | | | | |
| --- | --- | --- | --- | --- | --- | --- |
|  |  |  | **In 12 months prior to assault** | **Post assault^b^** |  |  |
|  |  | N^a^ | n(%) | n(%) | P value* | % diff |
|  |  |  |  |  |  |  |
| **Self Harm** |  | 65 | 26 (40.0%) | 33 (50.8%) | 0.210 | -10.8% |
|  |  |  |  |  |  |  |
| **Physical Symptoms** |  |  |  |  |  |  |
| Headaches |  | 67 | 40 (59.7%) | 48 (71.6%) | 0.152 | -11.9% |
| Abdominal pain |  | 62 | 28 (45.2%) | 38 (61.3%) | 0.052 | -16.1% |
| Poor sleep |  | 68 | 32 (47.1%) | 60 (88.2%) | **<0.000** | -41.2% |
| Changes in appetite |  | 65 | 18 (27.7%) | 49 (75.4%) | **<0.000** | -47.7% |
| Other physical symptoms |  | 61 | 1 (1.6%) | 4 (6.6%) | 0.375 | -4.9% |
| Any physical symptoms |  | 71 | 53 (74.6%) | 69 (97.2%) | **<0.000** | -22.5% |
|  |  |  |  |  |  |  |
| **Health service visit for physical symptoms** |  |  |  |  |  |  |
| Visited a general practitioner (GP) |  | 65 | 35 (53.8%) | 47 (72.3%) | **0.023** | -18.5% |
| Visited a hospital |  | 59 | 26 (44.1%) | 31 (52.5%) | 0.383 | -8.5% |
| Visited other service |  | 61 | 7 (11.5%) | 12 (19.7%) | 0.302 | -8.2% |
| Visited any service |  | 68 | 44 (64.7%) | 56 (82.4%) | **0.008** | -17.6% |
|  |  |  |  |  |  |  |
| **Health service visit for mental health symptoms** |  |  |  |  |  |  |
| GP |  | 61 | 12 (19.7%) | 12 (19.7%) | 1.000 | 0.0% |
| Mental Health Professional^c^ |  | 64 | 19 (29.7%) | 39 (60.9%) | **<0.000** | -31.3% |
| Counsellor^d^ |  | 67 | 19 (28.4%) | 29 (43.3%) | 0.076 | -14.9% |
| Other medical professional^e^ |  | 61 | 1 (1.6%) | 5 (8.2%) | 0.219 | -6.6% |
| Other service^f^ |  | 64 | 2 (3.1%) | 12 (18.8%) | **0.006** | -15.6% |
| Any service accessed for mental health help |  | 69 | 33 (47.8%) | 56 (81.2%) | **<0.000** | -33.3% |
| More than 1 service accessed |  | 73 | 19 (26.0%) | 29 (39.7%) | 0.087 | -13.7% |

^a^ N = number with valid data

^b^ Post assault: symptoms reported at first (4-5 months post sexual assault) and/or second follow-up (study end,13-15 months post sexual assault)

^c^ Mental health professional: Child and Adolescent Mental Health Services (CAMHS), family therapy, adult mental health services, psychologist, psychiatrist, psychotherapy. Includes in- and out-patient care

^d^ Counsellor: unspecified counselling, school counsellor, grief/bereavement counselling, group counselling, behavioural counselling, counselling for individuals who have suffered abuse

^e^ Other medical professional: Accident and Emergency (A&E), school nurse, gynaecology, sexual health clinic

^f^  Other service: Social services, school, voluntary sector support, advocacy, alternative therapies and unspecified support

*McNemar Test. P-values <0.05 were considered significant and are in bold

**Table C: Social outcomes following sexual assault among female participants**

|  |  | **Female participants followed-up to study end (N=71)** | | | | |
| --- | --- | --- | --- | --- | --- | --- |
|  |  |  | **Study entry** | **Study end** |  |  |
|  |  | N^a^ | n(%) | n(%) | P value* | % diff |
|  |  |  |  |  |  |  |
| **Re-victimisation** by study end (cumulative) |  | 69 | - | 10 (14.5%) | - | - |
|  |  |  |  |  |  |  |
| **Ever in foster care** |  |  |  |  |  |  |
| Prior to or at the time of the assault |  | 70 | 12 (17.1%) | - | - | - |
| By study end (cumulative) |  | 68 | - | 21 (30.9%) | - | - |
|  |  |  |  |  |  |  |
| **Education and employment** |  |  |  |  |  |  |
| All ages: In education or employment |  | 71 | 63 (88.7%) | 60 (84.5%) | 0.630 | 4.2% |
| All ages: Missed >30 days of school in last 12 months^b^ |  | 49 | 11 (22.4%) | 23 (46.9%) | **0.008** | -24.5% |
| Participants aged 13-15yr at *study entry* who were not in school |  | 71 | 0 | - | - | - |
| Participants aged 13-15yr at *study* *end* who were not in school |  | 22 | - | 3 (13.6%) | - | - |
| Participants aged 16yr+ at *study* *entry* who were not in education, employment or training (NEET) |  | 29 | 8 (27.6%) | 5 (17.2%) | 0.549 | 10.3% |
| Participants aged 16yr+ at *study end* who were not in education, employment or training (NEET) |  | 49 | - | 8 (16.3%) | - | - |
| All ages: Disengaged from education and employment^c^ |  | 71 | 22 (31.0%) | 30 (42.3%) | 0.152 | -11.3% |

^a^ N = number with valid data

^b^ Denominator is those in school

^c^ All those who were (i) in school but had missed >30 days of school in last 12 months or (ii) not in school (13-15y) or NEET (16y +)

* McNemar Test: P-values <0.05 were considered significant and are in bold
